# A Rare Presentation of Acute Cholecystitis Mimicking Malignancy in a Young Male With Elevated IgG4

**DOI:** 10.7759/cureus.110872

**Published:** 2026-06-15

**Authors:** Katherine Wong, Clara Wong

**Affiliations:** 1 Radiology, North District Hospital, Hong Kong, HKG; 2 Oncology, Yale Cancer Center, Yale University, New Haven, USA

**Keywords:** autoimmune cholecystitis, ct (computed tomography) imaging, hepatobiliary radiology, igg4 -related disease, usg imaging

## Abstract

A rare case of a 26-year-old male presenting with abdominal pain and jaundice was initially suspected to be a gallbladder malignancy based on imaging findings of a gallbladder fossa mass and biliary strictures. Serial imaging, including ultrasound, CT, PET-CT, and endoscopic retrograde cholangiopancreatography (ERCP), revealed a hypermetabolic mass with biliary obstruction but no gallstones. Elevated serum IgG4 levels and a marked clinical response to steroids led to the diagnosis of IgG4-related sclerosing cholangitis (IgG4-SC) and cholecystitis, an uncommon presentation that often mimics malignancy or acute cholecystitis.

This case highlights the diagnostic challenges and importance of considering IgG4-related disease (IgG4-RD) in young patients with inflammatory biliary masses and emphasizes multidisciplinary management to avoid unnecessary surgery.

## Introduction

IgG4-related disease (IgG4-RD) is a systemic fibroinflammatory condition characterized by tumefactive lesions, lymphoplasmacytic infiltrates rich in IgG4-positive plasma cells, and elevated serum IgG4 concentrations [1]. Autoimmune pancreatitis (AIP) is the most common manifestation, but IgG4-RD can also involve the biliary tree and gallbladder, causing sclerosing cholangitis and cholecystitis. These manifestations can closely mimic malignancies such as cholangiocarcinoma or gallbladder carcinoma, posing significant diagnostic difficulty [2]. Isolated IgG4-related cholecystitis and sclerosing cholangitis without pancreatic involvement are rare and often present with obstructive jaundice, biliary strictures, and mass-like lesions, leading to potential misdiagnosis and delayed treatment [3,4].

## Case presentation

A 25-year-old male presented to the Accident and Emergency Department with a two-day history of progressive jaundice, dark tea-coloured urine, pale yellow stools, and generalized malaise. He also reported right upper quadrant abdominal pain and low-grade fever. On clinical examination, he was septic, with mild tenderness over the right upper quadrant of the abdomen. No palpable abdominal mass or hepatosplenomegaly was noted. He had a past medical history of acute pancreatitis one year earlier, which subsequently resolved with antibiotics.

Initial laboratory investigations demonstrated a cholestatic pattern of liver dysfunction, with total bilirubin of 143 μmol/L, alkaline phosphatase (ALP) of 685 U/L, and alanine aminotransferase (ALT) of 152 U/L. The white cell count was within normal limits.

Investigation

Given these findings, an urgent abdominal ultrasound was performed, which showed an ill-defined, irregular, predominantly hypoechoic lesion (3.9 × 2.4 × 4.3 cm) with internal echogenic foci/calcifications in the liver hilum/gallbladder fossa, surrounding a collapsed gallbladder. No gallstones were identified (Figure 1).

**Figure 1:**
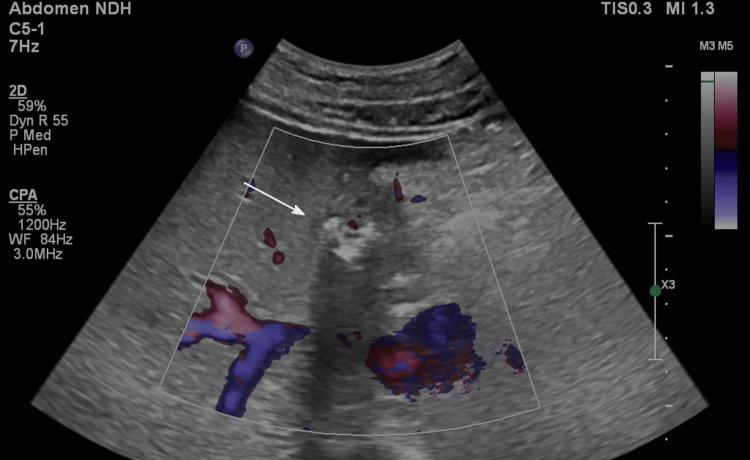
USG image of gallbladder The lesion (arrow) appears heterogeneous with hypoechoic areas interspersed with echogenic calcific foci and demonstrates internal vascularity on Doppler imaging.

An urgent CT abdomen was then performed on the same day, showing gallbladder wall thickening inseparable from the liver. The normal gallbladder was not visualized. At the gallbladder fossa, there was an ill-defined, complex, multiloculated, rim-enhancing mass insinuating along the porta hepatis region. It measured approximately 3.5 × 2.4 × 3.4 cm (AP × transverse × CC). Adjacent perilesional inflammatory stranding and fluid were present. The mass was inseparable from the inferior segment of the right hepatic lobe, where a hypoenhancing multiloculated area was identified. Dilated central intrahepatic ducts were also present (AP diameter of the right intrahepatic ducts: 5 mm; left: 4 mm). These features were concerning for acute-on-chronic cholecystitis with or without cholangitic liver abscess formation (differential diagnosis: neoplastic changes) (Figure 2).

**Figure 2:**
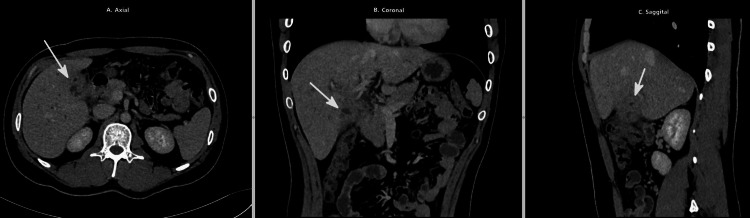
Contrast-enhanced CT of the abdomen with multiplanar reconstructions at the gallbladder level (A-C) Contrast-enhanced CT of the abdomen with multiplanar reconstructions (axial, coronal, sagittal), showing an ill-defined, complex, multiloculated, rim-enhancing mass insinuating along the porta hepatis region, with adjacent perilesional inflammatory stranding and fluid (arrows).

Given persistently deranged liver function tests, with elevated globulin (49 g/L), total bilirubin (34 μmol/L), ALP (963 U/L), and ALT (189 U/L), endoscopic retrograde cholangiopancreatography (ERCP) was performed. An interval ERCP was subsequently carried out, with placement of bilateral intrahepatic ductal stents to relieve biliary obstruction (Figure 3).

**Figure 3:**
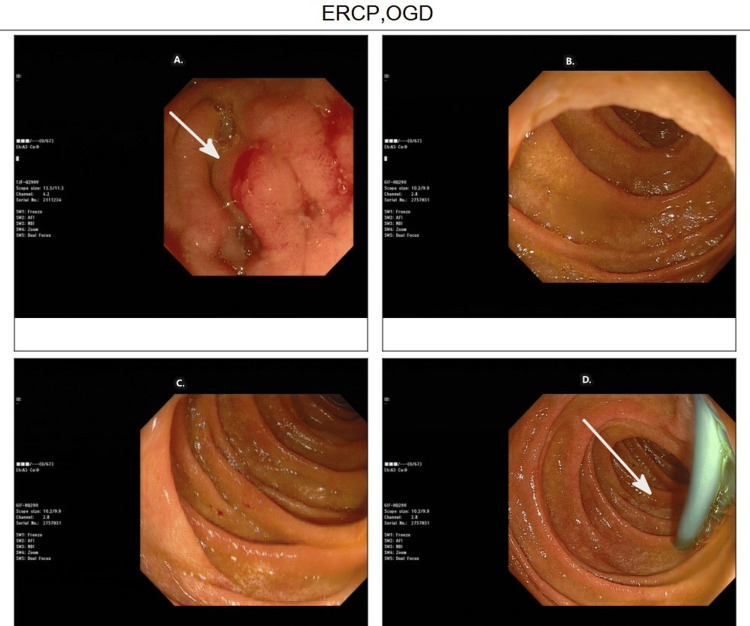
Endoscopic retrograde cholangiopancreatography (ERCP) and oesophagogastroduodenoscopy (OGD) images at the level of the ampulla of Vater (A-D) Snapshot images taken during endoscopic retrograde cholangiopancreatography (ERCP) and oesophagogastroduodenoscopy (OGD) (arrows). At the level of the ampulla of Vater, there are oedematous changes in the duodenal wall, with subsequent insertion of intrahepatic ductal stents.

PET-CT performed one week later showed two biliary ductal stents in situ. The bilateral intrahepatic ducts and common bile duct were not dilated secondary to ERCP. An irregular, hypermetabolic mass with heterogeneous enhancement was seen in the gallbladder fossa, with loss of the normal gallbladder configuration. There was loss of the fat plane between the mass, liver hilum, and adjacent structures; these features were worrisome for malignant invasion. Mild pericholecystic stranding, possibly neoplastic or inflammatory, was also noted. The findings were suspicious for gallbladder carcinoma or cholangiocarcinoma, whereas chronic cholecystitis was considered less likely in the absence of gallstones (Figure 4).

**Figure 4:**
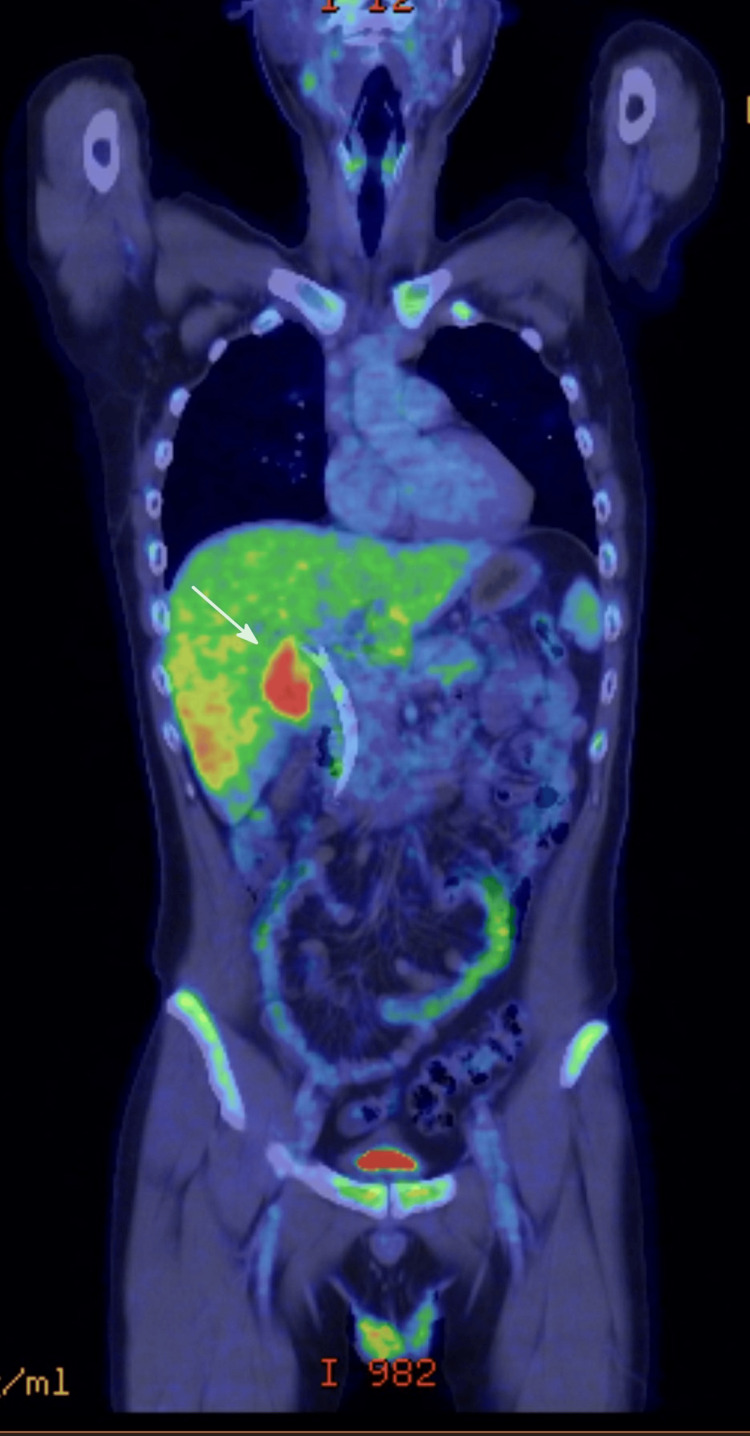
Fused FDG PET/CT coronal image showing a hypermetabolic lesion at the gallbladder fossa (arrow), with a corresponding SUVmax of 12.4

Histopathology from ERCP brushings was negative for malignancy, and the case was reviewed by the multidisciplinary team for further management. Following multidisciplinary discussion, serum IgG4 and CRP levels were obtained to guide treatment. Serum IgG4 was elevated (2.14 g/L). Empirical steroid therapy was initiated. If there was no clinical improvement, histological assessment with endoscopic ultrasound (EUS)-guided fine-needle aspiration (FNA) of the gallbladder lesion could be considered.

Following a six-week course of corticosteroid therapy, repeat CT imaging at three months demonstrated marked regression of the biliary lesion, strongly supporting the diagnosis of IgG4-related biliary disease (Figure 5).

**Figure 5:**
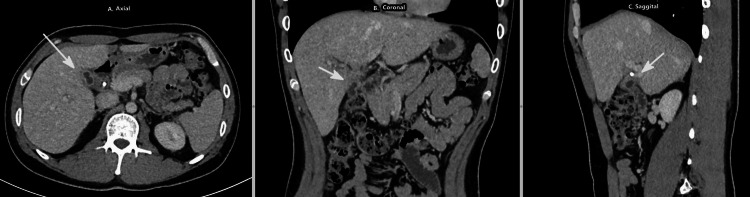
Follow-up contrast-enhanced CT (A-C) Follow-up contrast-enhanced CT of the abdomen with multiplanar reconstructions showing marked regression of the biliary lesion with normal configuration of the gallbladder (arrows).

## Discussion

IgG4-RD is a systemic fibroinflammatory condition characterized by infiltration of IgG4-positive plasma cells, storiform fibrosis, and often elevated serum IgG4 levels [1]. Although it may affect numerous organs, hepatobiliary involvement is diagnostically challenging, since clinical and imaging findings frequently overlap with malignancies such as cholangiocarcinoma or gallbladder carcinoma [2].

The most common manifestation in the hepatobiliary system is IgG4-related sclerosing cholangitis (IgG4-SC), often occurring in association with type 1 AIP [3,4]. Patients typically present with obstructive jaundice, abdominal pain, or weight loss, leading to initial concern for neoplastic disease [3]. Less frequently, isolated gallbladder involvement occurs, which may mimic acute-on-chronic cholecystitis but usually presents with gallbladder wall thickening or mass-like lesions in the absence of gallstones [5].

Radiological evaluation plays a central role in differentiating IgG4-RD from other pathologies. In the biliary tract, CT and MRI often demonstrate long, smooth strictures with wall thickening, in contrast to the short, irregular, or beaded strictures typical of primary sclerosing cholangitis [6]. Involvement of the gallbladder is characterized by mural thickening and enhancement, sometimes accompanied by adjacent inflammatory changes mimicking invasive carcinoma [7]. PET-CT may further complicate diagnosis due to hypermetabolic uptake, raising suspicion for malignancy [8].

Pancreatic involvement in IgG4-RD can resemble acute pancreatitis clinically and radiologically, with abdominal pain, elevated serum pancreatic enzymes, and inflammatory enlargement of the pancreas [9]. Similarly, IgG4-related cholecystitis may imitate acute calculous cholecystitis, but its hallmark feature is a dramatic response to corticosteroid therapy, reflecting an autoimmune aetiology rather than infection [5]. Given the close radiological and clinical similarities to cancer, a multidisciplinary diagnostic approach is imperative. This includes clinical evaluation, imaging, serological markers, histopathology, and steroid responsiveness [1,2,4]. Histopathology remains the diagnostic gold standard, though tissue sampling may be challenging.

Corticosteroids are the first-line therapy, inducing rapid clinical and radiological improvement in most patients [10]. For recurrent or refractory cases, immunosuppressive drugs such as azathioprine or biologic agents like rituximab offer alternative treatment strategies [10].

## Conclusions

This case highlights the diagnostic challenge of IgG4-related hepatobiliary disease, which can closely mimic malignancy or acute cholecystitis in young patients. Comprehensive evaluation, including imaging, elevated serum IgG4, histology, and response to corticosteroid therapy, is essential to avoid misdiagnosis and unnecessary surgery. Early recognition and treatment with steroids can lead to significant clinical and radiological improvement, underscoring the need for a multidisciplinary approach in managing these complex cases. IgG4-RD remains an important differential diagnosis in hepatobiliary masses and strictures, requiring awareness for timely intervention and better outcomes.

## References

[1] Kamisawa T, Zen Y, Pillai S, Stone JH (2015). IgG4-related disease. Lancet.

[2] Stone JH, Zen Y, Deshpande V (2012). IgG4-related disease. N Engl J Med.

[3] Nakazawa T, Naitoh I, Hayashi K, Miyabe K, Simizu S, Joh T (2013). Diagnosis of IgG4-related sclerosing cholangitis. World J Gastroenterol.

[4] Okazaki K, Uchida K, Ikeura T, Takaoka M (2013). Current concept and diagnosis of IgG4-related disease in the hepato-bilio-pancreatic system. J Gastroenterol.

[5] Ghazale A, Chari ST, Zhang L (2008). Immunoglobulin G4-associated cholangitis: clinical profile and response to therapy. Gastroenterology.

[6] Detlefsen S, Klöppel G (2016). Histopathology of IgG4-related disease (Article in German). Z Rheumatol.

[7] Zhang J, Chen H, Ma Y (2014). Characterizing IgG4-related disease with ¹⁸F-FDG PET/CT: a prospective cohort study. Eur J Nucl Med Mol Imaging.

[8] Hart PA, Zen Y, Chari ST (2015). Recent advances in autoimmune pancreatitis. Phil A Hart.

[9] Kamisawa T, Shimosegawa T, Okazaki K (2009). Standard steroid treatment for autoimmune pancreatitis. Gut.

[10] Khosroshahi A, Bloch DB, Deshpande V, Stone JH (2010). Rituximab therapy leads to rapid decline of serum IgG4 levels and prompt clinical improvement in IgG4-related systemic disease. Arthritis Rheum.

